# Mechanical Properties and Carbonation Durability of Engineered Cementitious Composites Reinforced by Polypropylene and Hydrophilic Polyvinyl Alcohol Fibers

**DOI:** 10.3390/ma11071147

**Published:** 2018-07-05

**Authors:** Wei Zhang, Chenglong Yin, Fuquan Ma, Zhiyi Huang

**Affiliations:** College of Civil Engineering and Architecture, Zhejiang University, 866 Yuhangtang Road, Hangzhou 310000, China; zhangw8778@zju.edu.cn (W.Z.); yinchenglong@zju.edu.cn (C.Y.); mafuquan@zju.edu.cn (F.M.)

**Keywords:** cost-efficiency, SHCC, ECC, PP fiber, hydrophilic PVA fiber, carbonation durability

## Abstract

Herein, the mechanical properties and carbonation durability of engineered cementitious composites (ECC) were studied. For the cost-efficient utilization of ECC materials, different types of specimens were cast with polypropylene (PP) and hydrophilic polyvinyl alcohol (HPVA) fibers. The compressive strength, Poisson’s ratio, strength-deflection curves, cracking/post-cracking strength, impact index, and tensile strain-stress curves of two types of ECC materials, with differing fiber contents of 0 vol %, 1 vol %, 1.5 vol %, and 2 vol %, were investigated with the use of compressive tests, four-point bending tests, drop-weight tests, and uniaxial tensile tests. In addition, the matrix microstructure and failure morphology of the fiber in the ECC materials were studied by scanning electron microscopy (SEM) analysis. Furthermore, carbonation tests and characterization of steel corrosion after carbonization were employed to study durability resistance. The results indicated that for both PP fiber- and HPVA fiber-reinforced ECCs, the compressive strength first increases and then decreases as fiber content increases from 0 vol % to 2 vol %, reaching a maximum at 1 vol % fiber content. The bending strength, deformation capacity, and impact resistance show significant improvement with increasing fiber content. The ECC material reinforced with 2 vol % PP fiber shows superior carbonized durability with a maximum carbonation depth of only 0.8 mm.

## 1. Introduction

The inherent quasi-brittle behavior of conventional cementitious materials produces a lower strain capacity and damage tolerance, leading to poor safety and low durability of infrastructures such as buildings, bridges, tunnels, etc. [1,2]. In recent years, a strain hardening cementitious composite (SHCC) containing high volumes of polymer fibers (up to 2 vol %) has been investigated and utilized for many applications, as it has been found to exhibit a pseudo strain-hardening ability under direct tensile stress [3,4,5]. In comparison to ordinary concrete, the main advantages of SHCC materials include significantly higher ductility and deformation capacity after initial cracking; therefore, SHCC materials possess a higher load-carrying ability, thus enhancing safety and durability for structures under catastrophic conditions [6,7].

Engineered cementitious composites (ECC) are the most typical type of SHCCs, and were first reported by Li et al., in 1994 [8,9]. The predominant theory for the design of ECC involves a cohesive crack-mechanics approach with two nondimensionalized parameters from steady-state cracking and multiple cracking (Li and Leung, 1992) [10]. ECC is well known for its strain-hardening ability and fine multi-cracking failure mode under uniaxial tension. Its tensile strain capacity can reach 3–5%, whereas the maximum crack width is restricted to only 60–100 μm [11,12]. Recently, a new kind of ECC material with a tensile strain capacity as large as 8% has been manufactured with randomly dispersed polyethylene (PE) fiber [13]. Therefore, the use of ECC could significantly improve the durability [14,15] of concrete components and structures, thereby reducing maintenance cost during their lifetime and meeting the demands of modern civil engineering.

The relatively high strain capacity and multiple cracking abilities of the ECC materials are heavily dependent on the fiber geometry, aspect ratio, volume fraction, etc. of the fibers [16,17]. In light of early developments, the two most commonly used polymer fibers in ECC materials are polyethylene (PE) and oiled polyvinyl alcohol fibers (PVA, Kuraray CO., LTD, Tokyo, Japan) [18,19]. However, the costs per unit volume of PE and PVA fiber are at least ten times higher than that of ordinary concrete, therefore increasing the cost of manufacturing and limiting the practical engineering applications of ECC. Thus, the development of cost-effective fiber-reinforced ECC materials is worthwhile [20]. One way of achieving this is to use hybrid fibers as toughening components. Zhang, et al. [21,22] investigated the mechanical properties of ECC reinforced by PVA-steel hybrid fiber and have shown that the individual crack width decreases significantly during the multi-cracking stage, and both the cracking and tensile strength increase with increasing steel fiber content. Pakravan et al. [12,23] studied the improvement in ductility of PVA and polypropylene (PP) hybrid fiber-reinforced ECC; the results of which indicated that both the flexural strength and ductility of the composites were higher than those of the non-reinforced material. An alternative solution is the use of cost-efficient PP fiber and hydrophilic PVA (HPVA) fiber in the matrix. Huang et al. [20] investigated the performance of PP-fiber-reinforced ECC (PP-ECC) beams under reversed cyclic loading. They showed that the energy dissipation capacity of the PP-ECC beam is 2.9-times higher than that of ordinary concrete and that less damage appeared in the PP-ECC beam due to the load carrying capacity of the PP fiber in the cement matrix. Sasmal [24] and Pan [15] considered the strong chemical bond between the fiber and matrix and found that due to the hydrophilicity of the unoiled PVA fiber, rupture during the multi-cracking stage was the limiting factor of strain capacity as opposed to pull-out failure. Furthermore, the amount of fiber content could increase flexural strength, bending ductility, and toughness.

The composition of ECC matrix is quite different from that of conventional concrete. Micromechanical analysis shows that the elimination of coarse aggregates produces a high cement content (2–3 times higher than that in ordinary concrete), and the large quantities of fine sand used in the matrix increase the cost of ECC. Therefore, the replacement of cement with fly ash and expanding the use of local ingredients are adopted for ECC design [25,26].

As a result, this study aimed to provide an ECC material designed using local ingredients and low-cost fibers. Extensive experimental tests of ECC materials with various volume fractions of PP fibers and HPVA fibers were used to evaluate its mechanical properties and carbonation durability. The development of cost-effective PP and HPVA reinforced ECCs are expected to support the design of ECC materials for various applications.

## 2. Materials and Methods

### 2.1. Material Properties

The use of local ingredients plays a major role in reducing cost and improving practical engineering applications of ECC [18,25]. Matrix materials employed herein included Portland cement (PC), fly ash (FA), silica sand, expansive agent, and polycarboxylate superplasticizer (PSP). Type-II 52.5-R Portland cement, manufactured by Conch Cement Co., Ltd., Wuhu, China, has a specific surface area of 365.3 m^2^/kg, an initial setting time of 123 min, and a final setting time of 181 min. Fly ash employed in this study is Class-F with low calcium content. The chemical compositions of both PC and FA are shown in Table 1. The particle size of silica sand ranges from 0 to 0.6 mm. Considering the latent higher shrinkage during hydration [27,28,29], it is necessary to mix an expansion agent in the matrix. Calcium sulfoaluminate (CSA), the expansion agent used herein, is manufactured by Tianjin BaoMing Co., Ltd., Tianjin, China. PSP is used to improve the flow properties of the mixture. 

**Figure 1:**
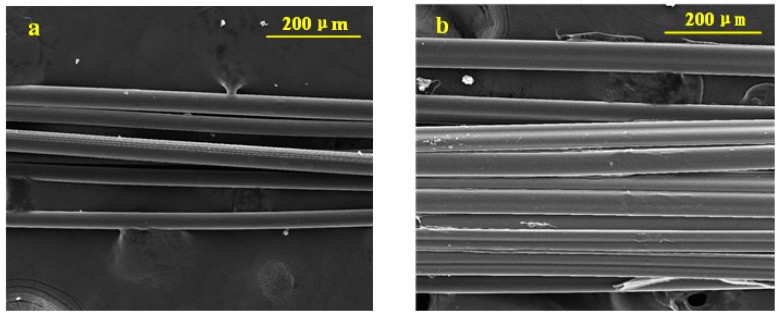
SEM images from: (**a**) PP fiber; and (**b**) HPVA fiber.

Two types of fibers were employed in this study: chopped polypropylene fibers (PP fiber) and hydrophilic polyvinyl alcohol fiber (HPVA fiber), supplied by Tianyi Company (Changzhou, China) and BHL Company (Quanzhou, China), respectively. The physical properties of the PP and HPVA fibers are given in Table 2, and the SEM images are given in Figure 1. 

### 2.2. Mix Design

In the current study, seven types of mixtures were cast, the composition details of which are listed in Table 3. Calculated amounts of CSA were added to the binders. Water-to-binder ratio was 0.3, silica sand-to-binder ratio was 0.4, and PSP occupied 0.2 wt % of total binders. The proportion of matrix was the same in all the samples. Fiber volume fractions (Vf), Vf = 1%, 1.5%, 2% of both PP fiber and hydrophilic PVA fiber were adopted and a plain cementitious composite (PCC) was prepared as a reference. Each mixture was demolded after 24 h at room temperature and cured for up to 28 days at a temperature of 23 °C ± 2 °C under a relative humidity of 95 ± 5%.

### 2.3. Experimental Work

The experimental program is divided into mechanical property tests and durability tests. In the first part, the effects of the types and contents of fiber on the compressive behavior, four-point bending performance, and drop-weight test are studied, and the typical tensile behavior of ECC with 2 vol % fiber is analyzed. In the durability test, the carbonation resistance of 2 vol % PP-fiber-reinforced ECC (PP-ECC) and the corrosion depth of rebar caused by carbonation are studied.

#### 2.3.1. Mechanical Property Tests

The compressive tests of PP-ECC and HPVA-ECC materials were performed by testing cylindrical-shaped specimens with dimensions Φ150 mm × 300 mm. Each group contained 3 samples, and a total of 21 cylinders were cast. Each cylinder was loaded into a pressure tester at 200 tons. Considering the errors caused by an uneven surface during casting, preloading was done with a rapid-hardening plaster in the cast surface to ensure a flat surface was achieved. The deformation curve was measured by two symmetrically arranged extensometers. A uniaxial load was applied at a rate of 0.5–0.8 MPa/s. Two vertical and two horizontal strain gauges were glued at mid-height of each sample for measuring the Poisson’s ratio.

**Figure 2:**
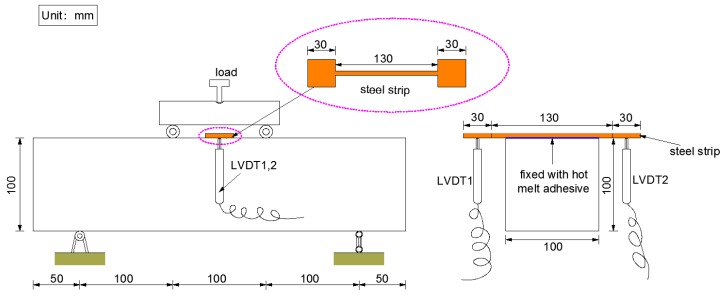
Details of four-point bending (4PB) tests.

Prismatic beams with dimensions of 100 mm × 100 mm × 400 mm were employed in the four-point bending (4PB) tests and a total of 18 specimens were cast. For measuring the mid-span deflections, a steel strip was fixed on the cross beams at the top position with a hot melt adhesive as a measuring point. The deflection values in the displacement control mode were measured at a rate of 0.1 mm/min by two symmetrically arranged linear variable differential transformers (LVDTs). The details of the 4PB tests are shown in Figure 2.

Impact resistance behavior was studied by the drop-weight test (ACI 544.2R-1999), dimensions of samples were Φ150 mm × 63 mm, and a total of 42 specimens were cast. Each specimen was impacted by a 4.5 kg drop hammer dropped from a height of 0.5 m to produce the impact energy.

Sheet-shaped specimens of dimensions 15 mm × 50 mm × 350 mm were subjected to uniaxial tensile tests [15], a total of 8 specimens were cast. Considering the resultant damage caused by stress concentration, a carbon fiber cloth and an aluminum plate were used to strengthen the ends of specimens with a length of 100 mm. The deflection of uniaxial tensile tests was measured by two symmetrically arranged LVDTs in deflection control mode at a rate of 0.1 mm/min.

#### 2.3.2. Durability Tests

**Figure 3:**
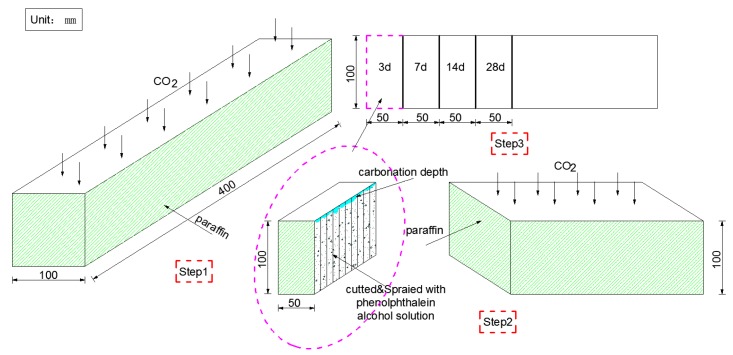
Details of the carbonization test.

The samples used in the carbonization test were beams with the dimensions 100 mm × 100 mm × 400 mm, a total of 3 specimens were cast. After curing for 28 d under standard conditions, five surfaces of each sample were sealed with paraffin and only one longitudinal surface was left untreated. When the carbonation test was performed, the concentration of CO_2_ gas in the carbonization chamber was controlled at 20 ± 3%. After erosion for 3 d, 7 d, 14 d, and 28 d, respectively, a 50 mm long section was cut off from one end of each sample and the new face of the remaining part was immediately waxed. Strike lines were drawn on the cut-off specimen every 10 mm and sprayed with 1% phenolphthalein alcohol solution (PAS) to measure the depth of carbonation based on the color change of PAS. The details of the carbonation test are shown in Figure 3.

**Figure 4:**
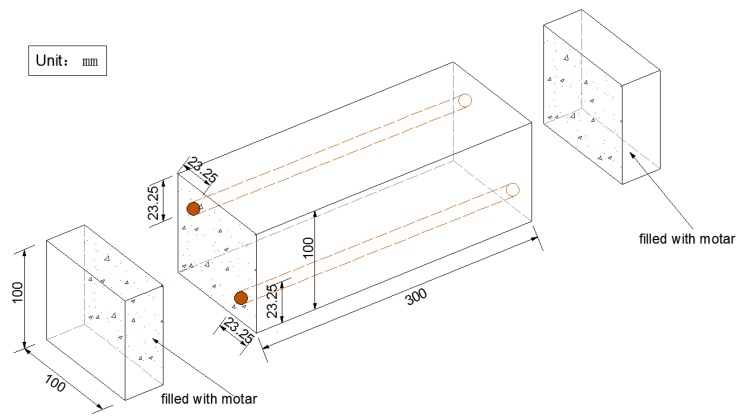
Details of the rebar corrosion test after carbonation.

Considering the corrosion of rebar due to the carbonization of concrete cover, which reduces durability of the structures, reinforcement corrosion after carbonation was investigated. Two rebars of diameter 6.5 mm and length 300 mm were embedded in the PP-ECC (Vf = 2%). After a curing period of 28 d under standard conditions, the specimens were placed in the carbonization chamber for another 28 d period to simulate a 50 year corrosion process under ordinary conditions. After this, each sample was cured under standard conditions for a further 28 days. Results of this test were obtained by measuring the weight loss of the rebars. For the weight loss measurement, each sample was broken in order to take out and weigh the rebars, which were then rusted and weighed after pickling. Considering the weight loss caused by the acid wash, two plain rebars were employed to account for this condition. Details of the reinforcement corrosion under carbonation are shown in Figure 4.

## 3. Result and Discussion

### 3.1. Mechanical Property Tests

**Figure 5:**
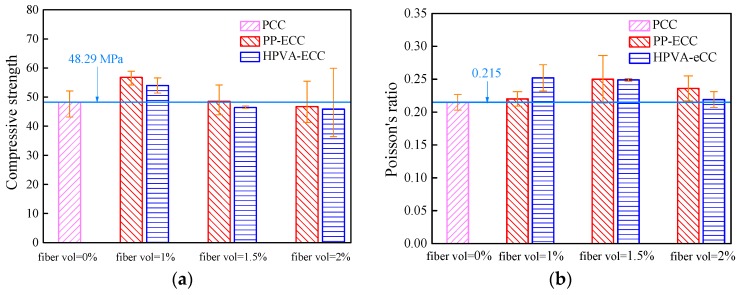
Results of compressive test: (**a**) compressive strength, and (**b**) Poisson’s ratio.

Compressive test: The compressive strength and Poisson’s ratio are reported as averages of the values obtained for three samples in each group. The relation of compressive strength and Poisson’s ratio with the types and volume fractions of fibers are given in Figure 5a,b, respectively. Error bars indicate the location of the average value between the maximum and minimum values. 

As shown in Figure 5a, the compressive strength of PCC is 48.29 MPa. After mixing with PP and HPVA fibers, the compressive strength increases up to a fiber content of 1 vol % and declines gradually for fiber contents greater than 1 vol %. This could be due to the potential of randomly distributed fibers to strengthen the matrix and control cracking propagation, thereby enhancing compressive strength [30]. However, when the fiber content exceeded 1 vol %, the high-volume fraction of fibers could not disperse evenly in the matrix, producing an increased amount of air bubbles and thus reducing the compressive strength [31]. Compared to the PCC, specimens with 1 vol % PP fiber and HPVA fiber showed an enhancement in the compressive strength by 17.50% and 11.82%, respectively, whereas the compressive strengths of those with 2 vol % fiber content decreased by ~3.71% and 5.09%, respectively. These results are similar to those reported by Pan et al. [32]. Moreover, fibers can control sliding and extending of micro-cracks, which limits lateral expansion and enhances Poisson’s ratio compared with PCC (Figure 5b).

Furthermore, as can be seen in Figure 5, for the same fiber content, all specimens mixed with PP fiber have a higher compressive strength than those which are HPVA fiber-reinforced. In general, composites with fibers possessing a high elastic modulus exhibit higher strengths [22,31,33]. In this study, although the modulus of HPVA fiber (42.8 GPa) is much higher than that of PP fiber (3.5 GPa), the opposite occurs due to the poor dispersivity of the HPVA fiber.

**Figure 6:**
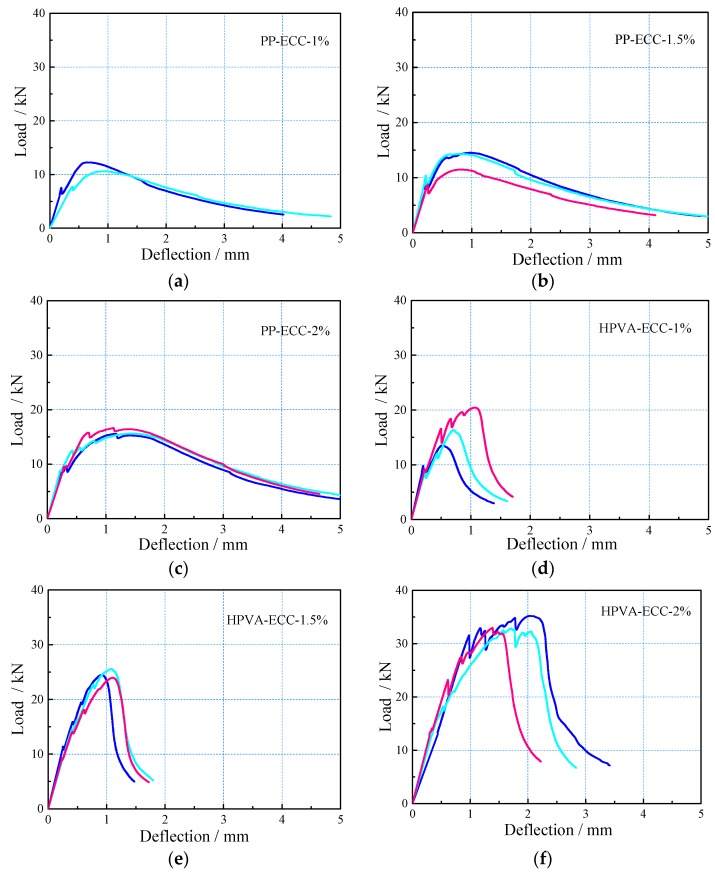
Load-deflection curves: (**a**) PP-ECC-1%, (**b**) PP-ECC-1.5%, (**c**) PP-ECC-2%, (**d**) HPVA-ECC-1%, (**e**) HPVA-ECC-1.5%, and (**f**) HPVA-ECC-2%.

4PB test: The strength-deflection curves for all the 4PB tests (Figure 6) show that each specimen possesses dramatically high ductility and load-deflection hardening ability under the 4PB test conditions. The shape of the load-deflection curves was highly influenced by the fiber types and contents, which is essential when choosing appropriate ECCs in bending structures of differing requirements. The area enclosed by the load-deflection curve increases with increasing fiber content, indicating that higher fiber fraction yields higher energy absorption capacity [23].

**Figure 7:**
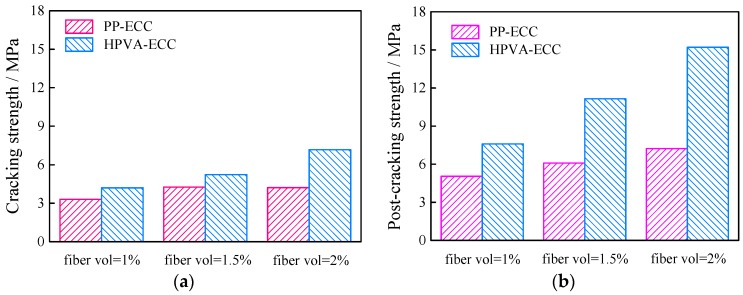
Results for (**a**) cracking strength, and (**b**) post-cracking strength.

Cracking and post-cracking strength also increased as a function of increasing fiber content (Figure 7). Composites reinforced with PP fiber show higher deformability but lower strength than those with PVA fibers for the same fiber content, which may be because PP fibers have a lower elastic modulus and tensile strength than HPVA fibers. Furthermore, by comparing the load-deflection curves of the two kinds of ECCs with the same fiber contents, it is found that specimens containing PP fibers show less softening response after post-cracking than those containing HPVA fibers. 

**Figure 8:**
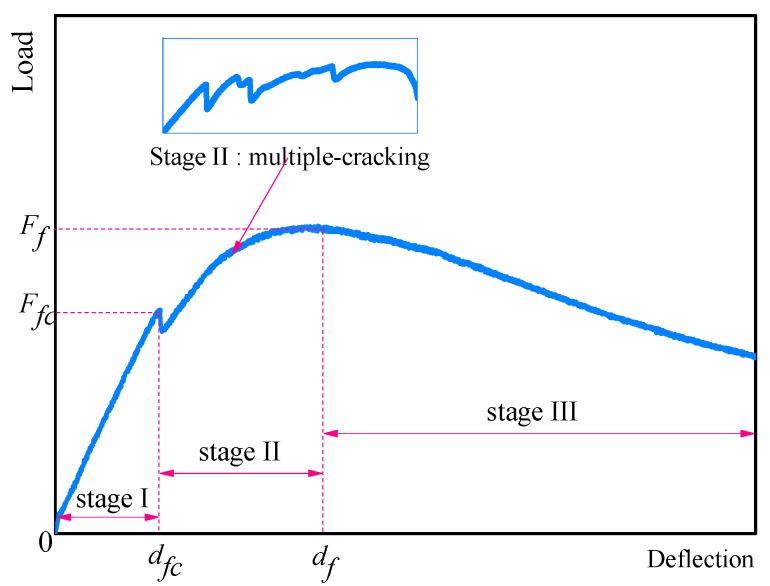
Typical bending load-deflection curves for ECC materials.

There are three distinct stages in a typical load-deflection curve of the ECC materials: Elastic stage, deflection-hardening stage, and deflection-softening stage (Figure 8).

Based on the research by Zhang et al. [22], we can define bending property as follows. During stage I, the relationship between load and deflection is that of a linear elastic until the first crack appears (*F*_fc_, and *d*_fc_ are the corresponding stress and deflection). For ECC materials with 2 vol % of PP fiber and 1–2 vol % of hydrophilic PVA fiber, stage II represents the load-deflection hardening characteristics accompanied by the multiple-cracking phenomenon. The post-cracking strength and the corresponding deflection are defined as *F*_f_ and *d*_f_, respectively. During stage III, a main crack is localized and the load starts to decrease gradually.

**Figure 9:**
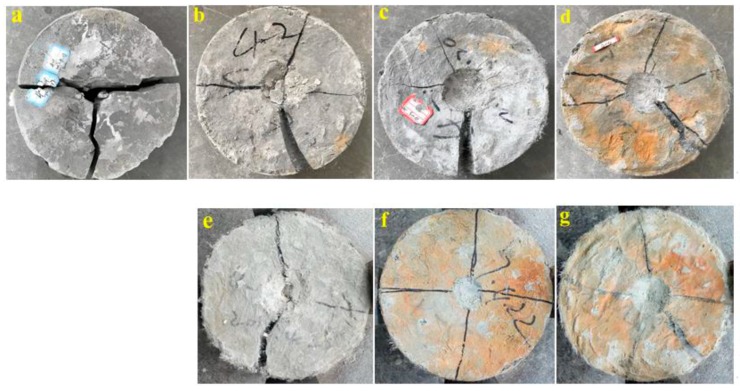
Typical fracture forms under drop weight test: (**a**) PCC, (**b**) PP-ECC-1%, (**c**) PP-ECC-1.5%, (**d**) PP-ECC-2%, (**e**) HPVA-ECC-1%, (**f**) HPVA-ECC-1.5%, and (**g**) HPVA-ECC-2%.

Impact resistance test: The impact times needed to produce the first crack (*N*_1_) and total failure (*N*_2_) in addition to the corresponding impact energies (*W*_1_ and *W*_2_) for specimens cured for 28 d are summarized in Table 4. The impact energy is determined by Equation (1). In addition, the typical failure forms of PP fiber- and PVA fiber-reinforced ECC with fiber fractions of 1 vol %, 1.5 vol %, and 2 vol % are shown in Figure 9.

*W*_i_ = *N*_i_*mgh* (1)

where *m* is the weight of the drop hammer (4.5 kg), *g* is the gravitational acceleration (9.81 m/s^2^), and *h* is the drop height (0.5 m). To determine the final values of *N*_1_ and *N*_2,_ each group should remove the maximum and minimum values and adopt the average of the remaining four values.

As can be seen in both Table 4 and Figure 9, PCC without fiber cracked after 8 hits and split into two or three fragments after only 13.75 hits. The corresponding impact energies were only 176.58 J and 303.5 J, respectively. The failure pattern shows clear brittle behavior. Conversely, after mixing with fiber to reinforce the cementitious composites, all the ECC specimens exhibited higher impact toughness than PCC. The impact times for first crack and failure increased significantly with increasing fiber content. For instance, when the PP fiber content increased from 1 vol % to 2 vol %, the impact times needed for crack and failure increased 3.6–3.9 times and 13.4–40.2 times, respectively. On the other hand, as the PVA fiber content increased from 1 vol % to 2 vol %, the impact times increased 18.3–160.7 times for first crack and 24.7–193.8 times for total failure. In addition, the type of fiber demonstrated a significant influence on the impact resistance. ECC specimens mixed with HPVA fiber have a much higher impact resistance than PP fiber-reinforced ones for the same fiber volume fraction. For instance, upon addition of 1 vol %, 1.5 vol %, and 2 vol % of PVA fiber, the impact times for crack and failure increased ~5.1/1.8 times, 13.9/2.8 times, and 41.5/4.8 times, respectively, compared with specimens containing the same volume fractions of PP fiber.

Figure 9 illustrates the typical impact failure form of specimens with different fiber types and contents. The failure mode of PCC shows apparent brittleness (Figure 9a). The first crack appears after an average of 8 hits and the main crack extends rapidly. Conversely, after mixing with PP and HPVA fiber, ECC materials exhibit apparent ductility under impact loading. For instance, upon mixing with less than 1 vol % of fiber, the main crack of ECCs propagates slowly due to fiber bridging. The typical failure mode of PP-1% and PVA-1% specimens was three splitting fractures, as shown in Figure 9b,e. Moreover, multi-crack formation was obtained by increasing fiber content, as shown in Figure 9c,d,f,g. Based on these results, ECC materials mixed with hydrophilic PVA fiber showed higher impact resistances than those mixed with PP fiber.

**Figure 10:**
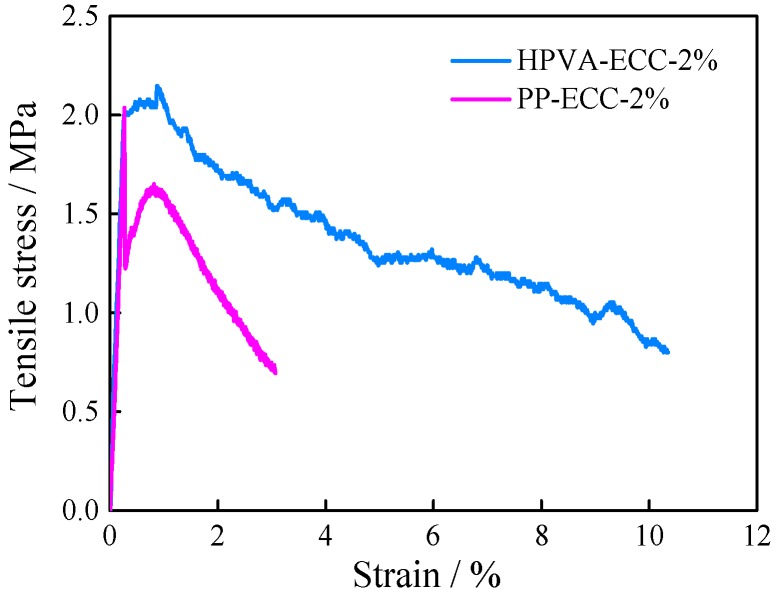
Tensile properties of PP-ECC and HPVA-ECC.

Uniaxial tensile test result: The fracture mode of ordinary concrete under tensile stress is brittle failure with the tensile strain-stress curves characterized by a linear elastic modulus up to the point of failure. Therefore, ordinary concrete displays a sudden break-down failure mode without warning. However, the main characteristic of ECC was strain-hardening after first cracking under tensile stress. Figure 10 illustrates the typical stress-strain curves of ECCs with 2 vol % PP fiber and 2 vol % HPVA fiber after curing for 28 days. 

Hydrophilicity of PVA fiber produces a strong chemical bond with the matrix, which should be considered for strain-hardening and multi-cracking [11]. However, the strain capacities of PP-ECC-2% and HPVA-ECC-2% were 0.83% and 0.88%, 83 times and 88 times larger than ordinary concrete (strain capacity of ~0.01%), while the cost is much lower than ECC with oiled PVA fiber and PE fiber (strain capacity of 3–5%).

**Figure 11:**
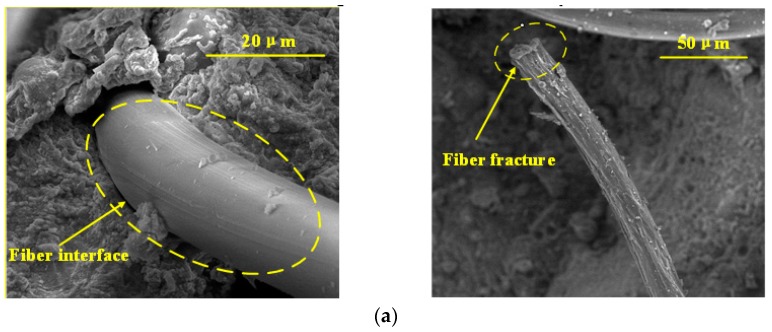

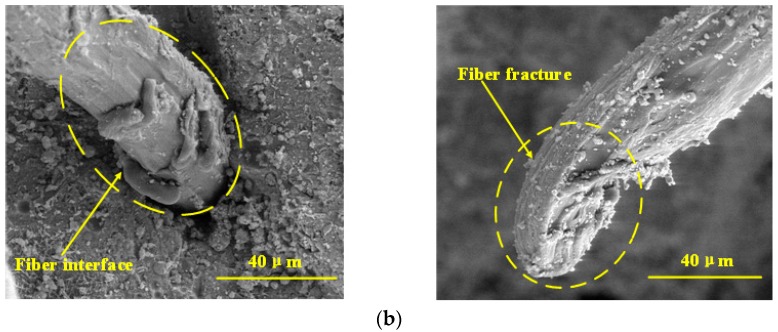
SEM images from: (**a**) PP fiber, and (**b**) HPVA fiber.

Selected microstructure analysis on the PP fiber and HPVA fiber in matrix after the tensile test is presented in Figure 11. The SEM images show that the PP fiber has a smooth clear surface and a smooth cross-section, while the HPVA fiber forms a strong chemical bond with the matrix as the fiber surface is covered by hydrated products. The main failure model of HPVA fiber is fracture, as opposed to pullout, which commonly occurred for oiled PVA.

### 3.2. Durability Ability

Carbonization test: Penetration of CO_2_ in cementitious materials led to a reduction in pH and accelerated the corrosion of rebars, resulting in a shorter lifetime of constructions, which is a major concern in terms of practical applications [34]. In this study, the average carbonation depth was derived from Equation (2).

(2) $$\bar{d_{t}}=\frac{1}{n}\overset{n}{\underset{i=1}{\sum }}d_{i}$$

**Figure 12:**
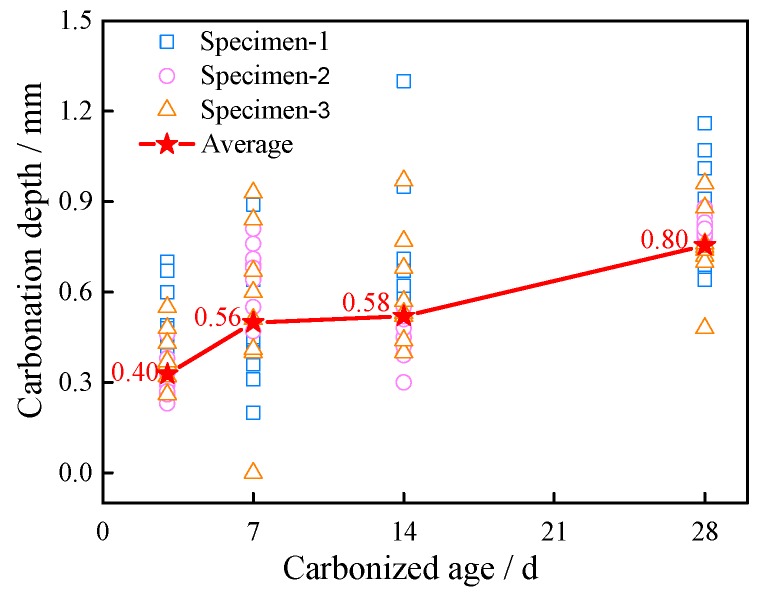
Carbonation depth of ECC specimens with 2 vol % PP fiber.

**Figure 13:**
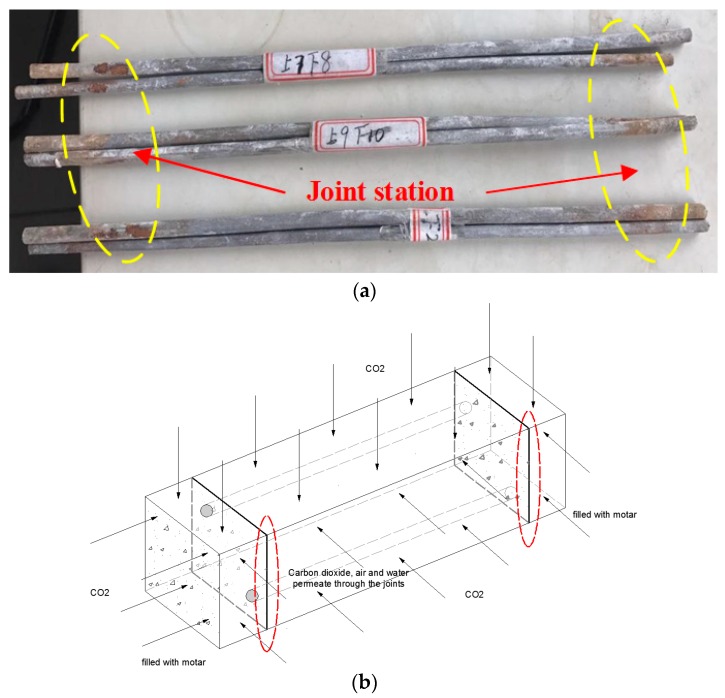
Carbonization corrosion tests of rebar for the ECC with 2 vol % PP fiber: (**a**) corrosion parts, and (**b**) erosion sketch.

Figure 12 presents the carbonation depth of ECC specimens containing 2 vol % PP fiber. Carbonation depth depends on the porosity and pore size distribution of the hardened ECC matrix [35]. In ECC specimens, elimination of coarse aggregates and the use of a small amount of fine sands result in low porosity but high drying shrinkage of the matrix. For example, the ultimate drying shrinkage of normal concrete is 400–600 μm/m [36], but conventional ECC can have 3 times that value under the same conditions. Some researchers have shown that PP fiber can reduce the depth of carbonation corrosion due to its cracking control ability [31]. As can be seen in Figure 13, the average corrosion depth is only 0.8 mm for a curing age of 28 days.

Carbonation corrosion test of rebar: Carbonation is one of the main factors leading to corrosion of reinforcement. The weight loss rate of rebar after corrosion under carbonation is determined herein. The influence on corroded rebar during pickling was also considered, and two plain rebars, named R01 and R02, were used as reference. The weight loss rate of rebar was derived according to Equation (3), and the results are shown in Table 5.

(3) $$L_{w}=\frac{\omega_{0}-\omega -\frac{\left(\omega_{01}-\omega_{1}\right)+\left(\omega_{02}-\omega_{2}\right)}{2}}{\omega_{0}}\times 100$$

where *L*_w_ is the weight loss rate of rebar (%); *ω*_0_ and *ω* are the weights of the rebar before and after erosion of carbonation; for reference rebar, *ω*_01_ and *ω*_02_ are the initial weights, and *ω*_1_ and *ω*_2_ are the weights after pickling.

As seen in Table 5, only 0.031–0.087% weight loss was obtained for rebar under carbonized corrosion. Figure 13 shows that corrosion only occurred when a combination of air, CO_2_, and water penetrated the weakness of the joints. Combined with the result of the carbonation test, this shows that PP-ECC has significant resistance to rebar carbonation, in addition to high carbonation durability.

## 4. Conclusions

This study investigated the mechanical properties and carbonation durability of ECC with various volume fractions of PP fibers and HPVA fibers. Based on the above discussion, the following conclusions can be drawn:

1. Cost-efficient ECC materials can be obtained by addition of PP fibers, HPVA fibers, and relatively coarse sand.
2. Compressive strength is increased upon increasing fiber content to 1 vol % but decreased slightly beyond that volume fraction due to the dispersivity and air content created in the matrix by the higher volume fraction of the fiber.
3. Bending performance and impact resistance are both significantly affected by the fiber types and fiber contents. In general, cracking strength, post-cracking strength, and initial/final impact resistance energy increased with increasing fiber contents, and ECC materials with HPVA fiber showed higher bending and impact resistance than those with PP fiber.
4. ECC materials with PP fiber and HPVA fiber show a lower strain capacity than those with oiled PVA fiber and PE fiber. However, low manufacturing costs make the ECC materials suitable for use.
5. Carbonation tests on PP-ECC with 2 vol % PP fiber revealed a carbonation depth of only 0.8 mm, which illustrates superior carbonation durability and greater protection for rebar over prolonged use.

## Figures and Tables

**Table 1:** Chemical compositions of Portland cement (PC) and fly ash (FA).

Materials	Chemical Compositions (Mass Fraction, %)							
	SiO_2_	Al_2_O_3_	Fe_2_O_3_	CaO	MgO	SO_3_	NaO	K_2_O
PC	9.68	3.63	3.91	50.59	1.55	1.45	0.12	0.39
FA	26.44	15.2	7.11	9.07	1.3	0.83	0.95	1.57

**Table 2:** Physical properties of polypropylene fibers (PP fiber) and hydrophilic polyvinyl alcohol fiber (HPVA fiber).

Name	Diameter (μm)	Tensile Strength (MPa)	Tensile Modulus (GPa)	Length (mm)	Density (g/cm^3^)	Elongation Percentage (%)
PP	16	≥500	3.5	12	0.91	15~20
HPVA	39	≥1600	42.8	12	1.3	6~8

**Table 3:** Mix properties of plain cementitious composite (PCC), PP-fiber-reinforced ECC (PP-ECC) and HPVA-fiber-reinforced ECC (HPVA-ECC).

Groups	Volume Fraction (%)		Weight Ratio of Matrix					
	PP	PVA	PC	FA	Water	Silica Sand	CSA	PSP
PCC	0	0	1	0.9	0.6	0.8	0.1	0.1%
PP-ECC-1%	1	0						
PP-ECC-1.5%	1.5	0						
PP-ECC-2%	2	0						
HPVA-ECC-1%	0	1						
HPVA-ECC-1.5%	0	1.5						
HPVA-ECC-2%	0	2						

**Table 4:** Impact resistance performance of PCC, PP-ECC and HPVA-ECC.

Group	*N*_1_ (Times)	*W*_1_ (J)	*N*_2_ (times)	*W*_2_ (J)
PCC	8	176.58	13.75	303.5
PP-ECC-1%	28.8	635.69	184.25	4066.86
PP-ECC-1.5%	29.5	651.14	428.5	9458.07
PP-ECC-2%	31	684.25	552.75	12,200.57
HPVA-ECC-1%	146	3222.59	339.75	7499.13
HPVA-ECC-1.5%	411	9071.8	1212	26,751.87
HPVA-ECC-2%	1285.5	283,374.2	2664.5	58,812.18

**Table 5:** Results of carbonization corrosion test of rebar for the ECC with 2 vol % PP fiber.

NO.	*ω*_0_	*ω*	*ω*_01_	*ω*_1_	*ω*_02_	*ω*_2_	*L*_w_ (%)
R_01_	-	-	55.516	55.142	-	-	-
R_02_	-	-	-	-	57.046	56.733	-
R_1_	49.085	48.711	-	-	-	-	0.062
R_2_	52.276	51.887	-	-	-	-	0.087
R_3_	55.691	55.312	-	-	-	-	0.064
R_4_	57.737	53.345	-	-	-	-	7.012
R_5_	50.668	50.289	-	-	-	-	0.070
R_6_	58.949	58.587	-	-	-	-	0.031
